# Using selected scenes from Brazilian films to teach about substance use disorders, within medical education

**DOI:** 10.1590/S1516-31802012000600005

**Published:** 2013-01-18

**Authors:** João Mauricio Castaldelli-Maia, Hercílio Pereira Oliveira, Arthur Guerra Andrade, Francisco Lotufo-Neto, Dinesh Bhugra

**Affiliations:** I MD. Preceptor, Discipline of Psychiatry, Faculdade de Medicina do ABC (FMABC), Santo André, São Paulo. Researcher in the Grupo Interdisciplinar de Estudos em Álcool e Drogas (GREA), Instituto de Psiquiatria, Faculdade de Medicina da Universidade de São Paulo (FMUSP), São Paulo, Brazil.; II MD, MSc. Psychiatrist, Department of Psychiatry, Faculdade de Medicina da Universidade de São Paulo (FMUSP), São Paulo, Brazil.; III MD, PhD. Full Professor, Department of Psychiatry, Faculdade de Medicina do ABC (FMABC), Santo André, São Paulo, and Associate Professor, Department of Psychiatry, Faculdade de Medicina da Universidade de São Paulo (FMUSP), São Paulo, Brazil.; IV MD, PhD. Associate Professor, Department of Psychiatry, Faculdade de Medicina da Universidade de São Paulo (FMUSP), São Paulo, Brazil.; V MA, MSc, MBBS, FRCP, FRCPsych, MPhil, PhD. Professor of Mental Health and Cultural Diversity, Institute of Psychiatry, King’s College, London, United Kingdom.

**Keywords:** Motion pictures as topic, Alcoholic intoxication, Substance-related disorders, Behavior, addictive, Education, medical, Cinema como assunto, Intoxicação alcoólica, Transtornos relacionados ao uso de substâncias, Comportamento aditivo, Educação médica

## Abstract

**CONTEXT AND OBJECTIVES:**

Themes like alcohol and drug abuse, relationship difficulties, psychoses, autism and personality dissociation disorders have been widely used in films. Psychiatry and psychiatric conditions in various cultural settings are increasingly taught using films. Many articles on cinema and psychiatry have been published but none have presented any methodology on how to select material. Here, the authors look at the portrayal of abusive use of alcohol and drugs during the Brazilian cinema revival period (1994 to 2008).

**DESIGN AND SETTING:**

Qualitative study at two universities in the state of São Paulo.

**METHODS:**

Scenes were selected from films available at rental stores and were analyzed using a specifically designed protocol. We assessed how realistic these scenes were and their applicability for teaching. One author selected 70 scenes from 50 films (graded for realism and teaching applicability ≥ 8). These were then rated by another two judges. Rating differences among the three judges were assessed using nonparametric tests (P < 0.001). Scenes with high scores (≥ 8) were defined as “quality scenes”.

**RESULTS:**

Thirty-nine scenes from 27 films were identified as “quality scenes”. Alcohol, cannabis, cocaine, hallucinogens and inhalants were included in these. Signs and symptoms of intoxication, abusive/harmful use and dependence were shown.

**CONCLUSIONS:**

We have produced rich teaching material for discussing psychopathology relating to alcohol and drug use that can be used both at undergraduate and at postgraduate level. Moreover, it could be seen that certain drug use behavioral patterns are deeply rooted in some Brazilian films and groups.

## RESUMO

**CONTEXTO E OBJETIVOS:**

Temas como o abuso de álcool e drogas, dificuldades nos relacionamentos, psicoses, autismo e transtornos de personalidade são largamente usados em filmes. Os filmes têm sido cada vez mais utilizados para ensinar psiquiatria e condições psiquiátricas em diferentes ambientes culturais. Existem muitos artigos sobre cinema e psiquiatria. Mas nenhum desses apresenta metodologia sobre como selecionar o material. Os autores se propuseram a buscar representação do uso problemático de álcool e drogas no período de retomada do cinema brasileiro (1994-2008).

**TIPO DE ESTUDO E LOCAL:**

Estudo qualitativo em duas universidades do estado de São Paulo.

**MÉTODOS:**

As cenas foram retiradas de filmes disponíveis em locadoras, e foram avaliados utilizando um protocolo desenvolvido especificamente. Foi avaliado o realismo e a aplicabilidade didática de cada cena. Um autor selecionou 70 cenas a partir de 50 filmes (notas de realismo e aplicabilidade didática ≥ 8) para posterior avaliação de outros dois autores. Diferenças nas notas dos três juízes foram avaliadas usando testes não-paramétricos (P < 0,001). Cenas com uma nota alta (≥ 8) foram definidas como “cenas de qualidade”.

**RESULTADOS:**

Trinta e nove cenas de 27 filmes foram identificadas como “cenas de qualidade”. Álcool, maconha, cocaína, alucinógenos e inalantes são mostrados nessas cenas. Sinais e sintomas de intoxicação, uso abusivo/nocivo e dependência foram encontrados.

**CONCLUSÕES:**

Foi produzido rico material para ensino, tanto na graduação quanto na pós-graduação, da psicopatologia relacionada ao uso de álcool e drogas. Além disso, foi possível demonstrar como alguns comportamentos relacionados ao uso de drogas estão arraigados em certos filmes e grupos brasileiros.

## INTRODUCTION

Films are a powerful medium and not only are influenced by the society and culture in which they are made but also influence society and culture in return. Use and abuse of psychoactive substances are a matter of concern not only in Brazilian society but also elsewhere. For any society, it is worthwhile investigating how substance abuse, dependence and addiction are portrayed in films and then, how this influences vulnerable members of the audience. It is also useful to explore how accurate the portrayal is and whether this is a true reflection of what is going on in society. This raises interesting questions for mental health professionals and trainers alike. Films are made for entertainment, but often there is a kernel of truth in what they portray and, therefore, it should be possible to use them for teaching medical students and psychiatric trainees.

Substance abuse is a result of several factors including genetic susceptibility and social factors such as peer pressure, wanting to belong to a subculture, excitement about the illegality of the act, expression of hostility, independence from parents and teachers, and reduction of unpleasant sensations.^1^

Widespread use of illicit drugs contributes to a range of social problems such as the possibility of increased crime and community violence; corruption of public servants; disintegration of social fabric of the society; emergence of new or complex health problems; lowering of productivity; ensnarement of youth in drug distribution and away from productive education or employment; and skewing of economies towards drug production and money laundering.^2^ Inevitably, substance misuse can exacerbate costs relating to medical care and within the family and personal spheres.^3^

Films have a potentially influential role, which already appears in other types of media.^4^ Films wrap the real world in a reel and then unroll it into a life of dreams and fantasy for their audiences. Films represent the author’s view but the interpretation is often that of the individual members of the audience, who see and interpret films in different ways. It is a type of art that provides an effective and powerful connection with the onlooker.^5^

In films, the themes of alcohol and drug abuse, difficulties in relationships, psychoses, autism and personality dissociation disorders are widely portrayed.^6^ This interaction is of major interest to mental health professionals because audiences get information from films. Psychoanalysts and psychotherapists not only appear more frequently in films than do psychiatrists, but also remain heavily engaged in analyzing cinema.^7^ Films about drugs are as old as cinema itself,^8^ starting in 1897 with *Chinese Opium Den*, by William Kennedy Dickson. Films have already portrayed the hedonism of substance users, with their social and medical impairments and the potential consequences, in quite a realistic fashion, such as in *Trainspotting* (1996), *Drugstore cowboy* (1989), *Rush* (1991), *Performance* (1970) and *London kills me* (1991).

Several medical schools around the world have integrated studies on the humanities into their undergraduate curricula, using philosophy, ethics, literature, theater and the arts.^9^ There is a strong trend in medical education towards insisting that any learning activity should contribute to the students’ development of concrete and measurable competencies, whether skills, knowledge or attitudes.^9^ Using films as a teaching tool can be a powerful means for engaging students, thereby clarifying their misconceptions and educating them about addiction medicine.^10^ Cultural and social factors play a key role in the way alcohol is used^11^ but often in relation to other substance use too. This can lead to use of appropriate and culturally sensitive methods for education and diagnosis. In Mediterranean countries, alcohol is introduced to young adults through wine, whereas in countries like the UK, binge drinking is common. Thus dealing with binge drinking will require different strategies. Earlier studies focused on American cinema,^12^^,^^13^^,^^14^ but such portrayals cannot be applied blindly to other cultures, although their impact can be studied within the cultural context. Therefore, specific issues relating to Brazil need to be explored separately.

Although Bhagar^15^ cautioned that there are no control studies relating to teaching and understanding mental illnesses through cinema, teaching of psychiatry using films has been shown to be a rewarding way of learning.^12^^,^^16^ Medical consultants are often used by film-makers to provide advice,^17^ thus making such films suitable for teaching.

It has been suggested that the younger generation learns in different ways and that new technologies should be used in medical education.^18^ The need for more research in this area has also been highlighted,^18^ given that use of films as teaching tools for psychiatry has not been thoroughly studied, especially in different cultures. Hyler et al.^19^ recommended using Hollywood portrayals of psychopathology, considering that there are no issues of patient confidentiality and that these films are made with high quality and entertainment values. Films have been successfully used for teaching pharmacology,^20^ medical professionalism^21^ and elements of the doctor-patient relationship.^22^

Perceptions of psychiatrists^23^ and electroconvulsive therapy (ECT)^24^ in films have changed over time. Akram et al.^23^ noted that the stigma relating to the psychiatry profession has decreased. However, there has been an increase in the case of ECT.^24^ Because individuals who use alcohol^25^^,^^26^ and drugs^26^ are potential victims of stigma applied by other people, and even from healthcare professionals in Brazil, selected scenes could be used to counteract stigma.

We set out to study the portrayal of substance and alcohol misuse in Brazilian cinema, which is an extremely rich source of material. Its richness comes not only from the great diversity of regional cultures, but also depictions of the “B side” of society in a more realistic fashion. This vocation arose in the Cinema Novo, Marginal or “de boca” tradition and has remained alive in the new generation of the so-called Revival of National Cinema. These new Brazilian directors acknowledge their admiration for their predecessors, from the Glauber Rocha generation, or from the popular films known as “chanchadas” and “pornochanchadas”.^27^

## OBJECTIVE

We decided to identify scenes with teaching potential in a systematic way, in order to use these to teach medical students the main concepts relating to alcohol and drug abuse and dependence, their diagnosis and their impact on others and on society.

## METHOD

### Design

We used a qualitative method to identify crucial scenes. It was decided to use a two-step approach: firstly to identify key scenes and then to obtain inter-rater agreement to check whether these scenes were suitable or not.

### Sample

We chose to focus on Brazilian films made during the Revival of National Cinema from 1994 to 2008, as these reflect not only a better picture of addictions but are also more recent and thus trainees are more likely to be aware of them. We decided to search among films that were available in DVD format and easily available for rental in stores.

We screened the labels to identify the subjects and the censor board classification of the films according to the themes. In addition, we also selected random films to ensure that these major themes had not being missed. Ease of availability was one of the key factors for the choice. Ten different rental companies in the cities of São Paulo and Campinas were used. Smoking was not included in this study due to the large number of scenes in which smoking is featured. Disorders relating to use of stimulants and tranquilizers were also not included.

### Inclusion criteria

- Brazilian (production or coproduction) film scenes;
- Portuguese-language film scenes (originally);
- Films made from 1994 onwards;
- Scenes that present any signs or symptoms of disorders relating to alcohol or drugs abuse classified in the International Classification of Diseases-10 (ICD-10) or the Diagnostic and Statistical Manual of Mental Disorders-IV (DSM-IV), translated;
- Scenes that present some behavior relating to use of alcohol or other drugs.

### Exclusion criteria

- Films from other countries and in other languages;
- Scenes produced up to 1993;
- Scenes in which signs, symptoms or behavior could not be clearly identified in relation to substance use (alcohol or other drugs).

### Assessment tools

#### A. Datasheet for evaluating the scenes

A datasheet was developed by three of the authors (JCM, AA and FLN) in order to evaluate and record scenes with alcohol and drug-related content. The key information recorded included the name of the film, synopsis, director, actors in the scene, the start and end times of the relevant scenes, signs and symptoms displayed, fulfillment of the ICD-10 or DSM-IV-TR criteria, type of disorders or symptoms (intoxication, abuse/harmful use, dependence, withdrawal etc.), type of substance shown (alcohol, cannabis, hypnotics, cocaine or hallucinogens; but not including tobacco), police involvement, psychodynamic factors involved, degree of realism (0-10), degree of teaching applicability (0-10), researcher and comments. Details and copies of the assessment datasheet can be obtained from the first author.

#### B. Rating scale for teaching applicability

A separate rating scale was developed to establish the best films for teaching the subject. On this scale, each relevant scene gained one point, each substance displayed gained one point, each related disorder found in ICD-10 gained one point, police involvement gained one point; and each scene that had a grade > 7.99 in relation to teaching applicability at the end of phase 2 gained two points. This scale was applied only to the films from which scenes were selected for phase 2 of the study (thus, the films selected were evaluated by the three judges).

### Procedure

All the films were initially studied by the first author, who completed the protocols for the scenes selected. A total of 192 scenes met the inclusion criteria and datasheets were filled out for these. One author (JMCM) selected all the scenes that had been qualified with two grades greater than or equal to 8 (68 scenes) for the next step. These selected scenes were sent for further analysis (new grading regarding how realistic the portrayal was and how useful the scene would be in terms of its teaching potential) to two other authors (HPO and FLN). Statistical analysis was performed on the grades awarded by these two observers. In this manner, high-quality scenes were identified and the films were then ranked according to their suitability for teaching. The first author drafted the manuscript and the other authors contributed to revisions.

### Statistical analysis

We used SPSS (Statistical Package for the Social Sciences) 18.0 for the analysis. Taking the grades to be discrete variables, we applied nonparametric tests (Friedman and Kendall W) for three dependent samples, in order to compare the three judges in relation to the ratings for realism and teaching applicability. For both realism (P = 0.001 in the two tests) and teaching applicability (P < 0.001 in the two tests), there were statistical differences among the three judges. A two-by-two nonparametric test (Wilcoxon and Sign) was applied to investigate these differences. In awarding grades, one evaluator was found to be different from the other two (P £ 0.01 in the two tests), but two evaluators did not differ (P = 0.37 in Wilcoxon and P = 0.76 in Sign). All three judges presented assessment differences regarding the teaching potential of the scenes (P < 0.001 in the two tests). Therefore, the scenes that had good averages (37 scenes) for the teaching potential grades (average > 7.99) were characterized as “quality scenes” for teaching. Thus, it was decided that “quality scenes” corresponded to a good average grade following evaluation by the three judges.

## RESULTS

**Figure 1.**
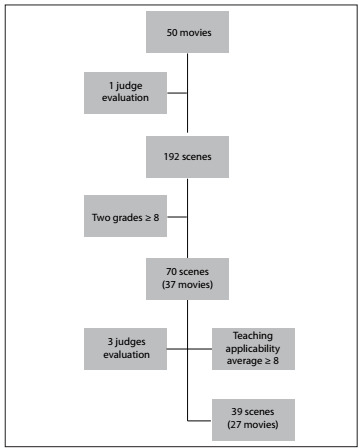
Algorithm illustrating the process of selecting the quality scenes.

In the first instance, a total of 192 scenes were identified from 50 films that were viewed **(**Figure 1**)**. The average number of scenes selected per film was 3.86. The films are listed alphabetically in Table 1. Over half (54%) of the films had a high-quality scene in terms of teaching applicability. Moreover, it is worth noting that 80% included one or more scenes relating to alcohol. Cocaine was shown in 38% of the films, followed by cannabis (36%). Table 1 also illustrates the scores on the rating scale for teaching applicability.

**Table 1.** Films evaluated

Films	Scenes	Substances	ICD 10	Police involvement	Teaching applicability Number of scenes/score
*A cartomante*	5	Alcohol Hallucinogens Hypnotics	F10.0/ F10.1/F10.2 F16.0/ F16.1 F13.1/F13.2	No	2 19
*A grande família*	4	Alcohol Hypnotics	F10.0/F10.1 F13.1	No	1 11
*Amarelo manga*	2	Alcohol	F10.0/F10.1	No	None
*Árido movie*	15	Alcohol Cannabis Hallucinogens Cocaine	F10.0/F10.1 F12.0/F12.2 F16.0	Yes	4 33
*Baixio das bestas*	5	Alcohol Cannabis	F10.0/F10.1 F12.0/F12.1	No	1 13
*Bellini e a esfinge*	2	Alcohol Cocaine	F10.0 F14.0/F14.2	No	None
*Bicho de sete cabeças*	5	Alcohol Cannabis Cocaine Hypnotics	F10.0/F10.1 F12.0 F14.0 F13.0/F13.1	Yes	1 18
*Bodas de papel*	6	Alcohol	F10.0	No	None
*Cafundó*	4	Alcohol Inhalants	F10.0	No	None
*Caixa dois*	2	Alcohol	F10.0	No	None
*Cama de gato*	3	Alcohol Hallucinogens Cocaine Cannabis	F10.0 F16.0 F14.0 F12.0	No	1 13
*Carandiru*	3	Cocaine Opioids	F14.0/F14.2 F11.0/F11.2?	Yes	3 16
*Cartola*	6	Alcohol	F10.0/F10.2	No	1 11
*Cazuza - o tempo não para*	5	Alcohol Cannabis Cocaine Hallucinogens	F10.0 F12.0 F14.0 F16.0	Yes	4 22
*O Cheiro do ralo*	5	Alcohol Cocaine	F10.0 F14.2	Yes	3 16
*Cidade baixa*	4	Alcohol Cannabis Cocaine	F10.0 F12.0 F14.0	No	1 12
*Cidade de deus*	6	Cannabis Cocaine	F12.0/F12.2 F14.0/F14.2	Yes	1 15
*Cidade dos homens*	3	Cannabis	F12.0	Yes	1 8
*Cinema, aspirinas e urubus*	4	Alcohol	F10.0/F10.2	No	1 9
*Contra todos*	*7*	*Alcohol* *Cocaine*	*F10.0* *F14.0*	*No*	*2* *15*
*Crime delicado*	2	Alcohol Hypnotics	F10.0 F13.0	No	None
*Dois perdidos numa noite suja*	2	Cocaine	F14.0/F14.2	No	1 7
*Estorvo*	5	Alcohol Cocaine	F10.0 F14.0	No	1 11
*Filme de amor*	2	Alcohol Cannabis Hypnotics Inhalants	F10.0 F12.0 F13.0 F18.0	No	None
*Inesquecível*	6	Alcohol	F10.0/F10.1	No	1 11
*Madame satã*	4	Alcohol Cocaine Inhalants	F10.0 F14.0 F18.0	Yes	None
*Meu nome não é Johnny*	7	Alcohol Cannabis Cocaine Inhalants	F10.0 F12.0/F12.1 F14.0/F14.1/F14.2 F18.0	Yes	1 21
*Muito gelo e dois dedos d´água*	2	Cannabis Opioids	F12.0 F11.0	Yes	None
*Não por acaso*	2	Alcohol	F10.0	No	1 6
*Narradores de Javé*	2	Alcohol	F10.0/F10.1	No	1 7
*Nina*	3	Hallucinogens Cannabis	F16.0/F16.1? F12.0	No	1 10
*No meio da rua*	1	Cannabis	F12.0	Yes	None
*O casamento de Romeu e Julieta*	4	Alcohol	F10.0	No	None
*O céu de Suelly*	4	Alcohol Inhalants	F10.0 F18.0	No	None
*O homem do ano*	3	Alcohol Cocaine	F10.0 F14.0/F14.1/F14.2	No	None
*O homem que desafiou o diabo*	5	Alcohol	F10.0/F10.2	No	1 10
*O invasor*	2	Alcohol Cannabis Cocaine Hallucinogens	F10.0/F10.1 F12.0/F12.1 F14.0/F14.1 F16.0/F16.1	No	1 16
*O maior amor do mundo*	4	Alcohol Cannabis Cocaine	F10.0 F12.0	Yes	1 12
*Ó pai, ó*	3	Alcohol Inhalants	F10.0/F10.1 F16.0	No	None
*Ônibus 174*	1	Inhalants	F16.0	No	None
*Os doze trabalhos*	1	Cannabis		No	None
*Os amadores*	2	Alcohol	F10.0	No	None
*As procuradas*	7	Alcohol Hypnotics	F10.0/F10.1 F13.0/F13.1	No	None
*Quase dois irmãos*	3	Cannabis Cocaine	F12.0	Yes	None
*Saneamento básico*	2	Alcohol		No	None
*Se eu fosse você*	4	Alcohol	F10.0	No	1 8
*Sonhos e desejos*	3	Alcohol	F10.0	No	None
*Tropa de elite*	3	Alcohol Cannabis Cocaine	F10.0 F12.0 F14.0	Yes	None
*Tudo isto é fado*	6	Alcohol	F10.0	Yes	1 11
*Zuzu Angel*	3	Alcohol	F10.0	No	None

*In bold: top ten films on the teaching applicability rating scale; ICD-10 = International Classification of Diseases-10.

The ICD-10 and/or DSM-IV-TR criteria were met in at least one selected scene in 96% of the films. Among the scenes, 78% met some of the diagnostic criteria for the disorders listed in the manuals. Not surprisingly, the criteria for intoxication were the ones most commonly met (74% of scenes), followed by criteria for abuse/harmful use (11%) and dependence (10%). Other disorders relating to use of alcohol and psychoactive substances (abstinence, withdrawal with delirium, psychotic disorder, amnesic syndrome, residual psychotic disorder, late-onset psychotic disorder, other mental and behavioral disorders and unspecified mental and behavioral disorders) were not shown in these films. Table 1 illustrates that the only relevant chapter of the ICD (among Chapters F10-19) that was not present in the study findings was the one relating to stimulants (F15).

The only diagnostic classification that generated a conflict between the two manuals (ICD 10 and DSM-IV-TR) was the harmful use and the abuse of psychoactive substances, respectively. Police involvement was a target for evaluation, but it was only found in 28% of the films selected and in 10% of the scenes. Physical violence was present in 17% of the scenes. Not surprisingly, these Brazilian films portray aspects of Brazilian culture such as the habit of drinking before meals, which is a very common occurrence. Thus, these films reflect the true position and reality.

Various psychodynamic factors relating to use of alcohol and other substances appeared in 59% of the scenes. The most common ones were: (1) coping with anxiety and stress; (2) coping with traumatic events; (3) celebration of important dates and personal victories; (4) building up courage to act; (5) dating; (6) coping with tedious daily life; (7) helping to express emotions and secrets; and (8) desire or pressure to feel new sensations.

The results from statistical analysis on the grades given by the three judges are presented in Tables 2**and**3.

**Table 2.** Results from nonparametric tests (Friedman and Kendall) applied to three dependent samples, in order to compare the three judges in relation to the ratings for realism and teaching applicability

	Realism				Teaching applicability			
	Mean	SD	P (Friedman)	P (Kendall)	Mean	SD	P (Friedman)	P (Kendall)
Judge 1	9.24	0.69	< 0.01	< 0.01	8.54	0.76	< 0.01	< 0.01
Judge 2	9.13	0.92			9.13	0.87		
Judge 3	9.21	1.77			6.62	2.47		

**Table 3.** Results from two-by-two nonparametric test (Wilcoxon and Sign) applied to investigate differences between judges

	Realism							
	Rank type	n	Mean ranks	Sum of ranks	Z (Wilcoxon)	P (Wilcoxon)	Z (Sign)	P (Sign)
Judge 2	Negative	24 (a)	24.63	591	- 0.892	0.37	- 0.292	0.76
versus	Positive	21 (b)	21.14	444	*			
Judge 1	Tie	23 (c)						
	Total	68						
Judge 3	Negative	34 (d)	30.26	1029	- 4.204	< 0.01	-2.571	0.01
versus	Positive	15 (e)	13.07	196	*			
Judge 1	Tie	19 (f)						
	Total	68						
Judge 3	Negative	34 (g)	23.06	784	- 3.792	< 0.01	-3.66	< 0.01
versus	Positive	9 (h)	18.00	162	*			
Judge 2	Tie	25 (i)						
	Total	68						
	Teaching applicability							
	Rank type	n	Mean ranks	Sum of ranks	Z (Wilcoxon)	P (Wilcoxon)	Z (Sign)	P (Sign)
Judge 2	Negative	7 (a)	27.36	191.5	- 3.818	< 0.01	- 4.472	< 0.01
versus	Positive	38 (b)	22.20	843.5	^†^			
Judge 1	Tie	23 (c)						
	Total	68						
Judge 3	Negative	47 (d)	31.50	1480.5	- 5.229	< 0.01	- 4.768	< 0.01
versus	Positive	10 (e)	17.25	172.5	*			
Judge 1	Tie	11 (f)						
	Total	68						
Judge 3	Negative	51 (g)	31.06	1584	- 6.044	< 0.01	- 5.828	< 0.01
versus	Positive	6 (h)	11.50	69	*			
Judge 2	Tie	11 (i)						
	Total	68						

(a) Judge 2 < Judge 1; (b) Judge 2 > Judge 1; (c) Judge 2 = Judge 1; (d) Judge 3 < Judge 1; (e) Judge 3 > Judge 1; (f) Judge 3 = Judge 1; (g) Judge 3 < Judge 2; (h) Judge 3 > Judge 2; (i) Judge 3 = Judge 2.

*Based on positive ranks; ^†^Based on negative ranks

The films containing scenes that were classified as quality scenes with regard to their degree of realism and/or extent of teaching applicability are described in Table 4. This table also shows the start and end time of each scene, as well as the substance depicted and the ICD-10 diagnosis that is suggested by the performance.

**Table 4.** Quality film scenes with regard to teaching applicability (Grade > 7.99)

Films	Beginning	End	Substances	ICD 10
*A cartomante*	1’58	10’52	Alcohol	F10.0/F10.1
			Hallucinogens	F16.0/F16.1
	56’00	1h 3’42	Alcohol	F10.0
*A grande família*	47’36	51’36	Alcohol	F10.0
*Árido movie*	20’15	22’50	Cannabis	F12.0
	49’53	58’34	Alcohol	F10.0
			Cannabis	F12.0
	1h 26’30	1h 29’19	Cannabis	F12.0
	1h 29’19	1h 38’01	Hallucinogens	F16.0
*Baixio das bestas*	46’00	48’53	Alcohol	F10.0
*Bicho-de-sete-cabeças*	4’16	8’04	Cannabis	F12.0
*Cama de gato*			Alcohol	F10.0
			Cannabis	F12.0
			Cocaine	F14.0
	12’06	17’07	Hallucinogens	F16.0
*Carandiru*	31’50	33’47	Cocaine	F14.0/F14.2
	44’52	47’09	Opioids	F11.0/F11.2?
	1h 34’43	1h 36’26	Cocaine	F14.0
*Cartola*	1h 19’00	1h 20’55	Alcohol	F10.0
			Alcohol	F10.0/F10.1
*Cazuza, o tempo não pára*	5’44	8’54	Cannabis	F12.0/F12.1
	26’48	28’17	Alcohol	F10.0/F10.1?
	29’29	31’17	Cannabis	F12.0
			Alcohol	F10.0
			Cocaine	F14.0
	31’18	35’02	Hallucinogens	F16.0
*O cheiro do ralo*	11’36	12’39	Cocaine	F14.2
	33’21	34’53	Cocaine	F14.2
	1h 4’17	1h 5’55	Cocaine	F14.2
*Cidade baixa*	6’26	12’06	Alcohol	F10.0/F10.1?
*Cidade de Deus*	49’56	51’30	Cannabis	F12.0
*Cidade dos homens*	1h 8’04	1h 11’32	Cannabis	F12.0
*Cinema, aspirinas e urubus*	1h 19’26	1h 26’16	Alcohol	F10.0
*Contra todos*	51’41	56’25	Alcohol	F10.0
	1h 29’47	1h 31’43	Cocaine	F14.0/F14.2?
*Dois perdidos numa noite suja*	1h 08’48	1h 13’33	Cocaine	F14.0
*Estorvo*	16’12	19’45	Alcohol	F10.0/F10.2
*Inesquecível*	43’04	51’22	Alcohol	F10.0/F10.1
*Meu nome não é Johnny*	28’08	31’02	Alcohol	F10.0/F10.1
			Cocaine	F14.0/F14.1
			Inhalants	F18.0/F18.1
*Não por acaso*	1h 5’05	1h 7’42	Alcohol	F10.0
*Narradores de Javé*	47’58	50’43	Alcohol	F10.0
*Nina*	47’24	48’42	Unknown	F1x.0/F1x.1
*O homem que desafiou o diabo*	4’17	9’37	Alcohol	F10.0
*O invasor*	38’15	52’33	Alcohol	F10.0/F10.1
			Cannabis	F12.0/F12.1
			Cocaine	F14.0/F14.1
*O maior amor do mundo*	53’49	59’03	Cannabis	F12.0
*Se eu fosse você*	16’36	18’50	Alcohol	F10.0/F10.1?
*Tudo isto é fado*	11’39	12’52	Alcohol	F10.0

### Alcohol

Many films present interesting material for observation of people making use of alcohol. *Baixio das bestas (Bog of beasts),* by Claudio Assis, has two major scenes. In the first scene, the role of alcohol in mood swings, increased aggression and eroticism is seen in several characters. They engage in an orgy in a brothel, with elements of physical sexual aggression. In the second scene, almost all the symptoms relating to alcohol intoxication are seen in a character who gathers the courage to declare his love to his beloved’s grandfather. It is interesting to note the fleeting nature of that courage, because the man soon gives in to the first “dressing down” by the girl’s grandfather.

Other films of interest in discussing alcohol use are: *Cartola*; *Madame satã*; *O homem que desafiou o diabo*; and *Cafundó (Cartola, Madame satan, The man who defied the devil* and *Cafundó)*. It is worth noting that alcohol use often appears together with scenes showing use of other substances as well.

### Opioids

We could only find two scenes of opioid use among the 192 scenes that we catalogued. In the film *Carandiru*, the actor Lázaro Ramos plays a character who, in order to get an injection drug (heroin) while in jail, commits several dangerous acts thus suggesting a dependency pattern.

### Cannabis

Watching the film *Árido movie* (*Arid movie*) in its entirety is perhaps the best way to discuss the psychopathology and developments relating to cannabis use. This film allows discussion of some of the positive and negative aspects of cannabis use, given that the effects experienced by the characters vary greatly during the scenes. There is violence and police involvement, but there are also scenes of heightened sense of pleasure and relaxation, sometimes with concomitant use of alcohol.

In *O maior amor do mundo* (*The greatest love of all*) both negative and positive aspects of cannabis use can also be identified. The young boy uses the substance and is involved with drug trafficking in the shantytown in the role of “airplane” (*messenger*). The actor José Wilker stars in a beautiful scene of intoxication accompanied by the boy. He experiences a feeling of great pleasure at being intoxicated at an extremely difficult time in his life.

### Sedatives and hypnotics

There were many scenes that showed sedative use. In most cases, the scenes included concomitant use of other substances (mainly alcohol), which would classify them as instances of abuse in the DSM-IV-TR. Only a few scenes showed symptoms of intoxication, even if to a mild degree. Most showed consumption only in situations of psychological stress, as in the scene in *A cartomante (The fortune teller)*, starring the actor Ilya São Paulo, when he discovers that he is being betrayed by his best friend. Another interesting scene occurs in *Bicho de sete-cabeças (Brainstorm)*, where the chief doctor of the asylum uses a sedative pill for himself, along with whisky. In this, the cumulative effects (symptoms) of intoxication due to these two substances can be seen. The psychodynamic aspect of this consumption consisted of relief or affective anesthesia in the situation. The character seeks relief because he witnesses horrifying events within day-to-day life in the asylum system.

### Cocaine

A greater variety of disorders was depicted in relation to cocaine, perhaps because this substance has the most negative scenes with its usage. In *O cheiro do ralo* (*Drained*), there are three scenes that scored high in realism in showing the likely path of a crack/cocaine dependent (the scenes do not show explicit consumption but it is implied through behavior), who begins to sell items from his family home in order to afford cocaine consumption, and who eventually sells his own nakedness to make more money. In the film *O homem do ano* (*The man of the year*), Murilo Benício plays a man with strong antisocial traits, which are enhanced during his first use of cocaine, which he takes in binges. After using cocaine, the character gains the courage to carry out revenge against an enemy who killed a friend and his pet. In *Dois perdidos numa noite suja* (*Two lost people in a dirty night*), Débora Falabella plays the role of a crack dependent who lives in the United States. She sustains her addiction by selling oral sex while disguised as a boy. In this film, a very well-produced scene of intoxication that includes development of all the symptoms is seen. Such a scene allows discussion about dependence and use of prostitution to obtain cocaine.

### Hallucinogens

In *Árido movie* (*Arid movie*), there is a vivid scene starring Guilherme Weber and José Dumont, in which the latter invites the former to try some tea made of unidentified roots, at a time of great stress for the character. The changes in sensory perception caused by the tea are depicted in a very realistic fashion.

### Volatile solvents

There are a few scenes involving use of these substances by children who are outside of the school environment. One scene that was selected takes place in *Ônibus 174 (Bus 174*), in which two pre-teenage children sniff shoemaker’s glue and experience symptoms of intoxication. Another type of common use can be seen in *Ó pai ó (Look at that)* by Monica Gardenberg, in which adults use inhalants during the first day of carnival in Salvador.

### Multiple drugs

Several scenes depicted intoxication by two or more substances, but two scenes are extraordinary in their depiction, especially regarding psychopathology In *Meu nome não é Johnny* (*My name is not Johnny)*, the leading actor Selton Mello is at a party that he hosts, and he uses three types of substances: alcohol, cocaine and inhalants. Consequently, he experiences symptoms of empowerment, elated mood, relaxation, loss of balance and changes in sensory perception. This scene received the highest grade. In *Cazuza, o tempo não pára (Cazuza),* the symptoms are not centered on one character alone, but on a group, following use of alcohol, cocaine and hallucinogens (many concurrently), within the context of a concert by the rock band *Barão Vermelho.*

### Treatment

The most famous of the Brazilian films dealing with psychiatric treatment is *Bicho de sete cabeças (Brainstorm),* by Laís Bodanzky. Rodrigo Santoro plays the part of a young man whose drug misuse condition is exacerbated after incorrect intervention. The patient is involuntarily admitted, without any real indication for this type of treatment (he had never received outpatient treatment, and showed no signs of mind-altering effects). He passes through various asylum-type institutions, and eventually receives ECT, more as a form of punishment than as a treatment.

In another film, *Meu nome não é Johnny* (*My name is not Johnny*), the character Johnny is arrested for drug trafficking and is referred for treatment in the House of Detention and Psychiatric Treatment. Although once again portraying a model of treatment in the asylum style, where the character goes through many hardships, an improvement in his condition can be seen, in that he manages to stay abstinent after treatment.

## DISCUSSION

The main purpose of this study was to show how material suitable for teaching important aspects of psychiatry could be selected for use, especially in relation to alcohol and drugs in Brazil. This material can thus be used to teach not only psychiatric trainees but also medical students and other professionals such as nurses, social workers, psychologists and general practitioners.

We were able to cover seven of the nine chapters in the ICD-10. Among the scenes that were classified as quality scenes regarding their teaching applicability, we found scenes showing disorders relating to use of alcohol, opiates, cannabis, cocaine/crack, hallucinogens, inhalants and many other drugs. Regarding types of disorder, more than one quality scene was found for each of the top three (intoxication, abuse/harmful use and dependence).

Recent studies have reported on the applicability of films for teaching medical students about psychiatry.^12^^,^^23^ However, the few previous studies that have evaluated films as a teaching tool were small and non-systematic,^12^^,^^15^^,^^28^ thus differing from ours. Here, we tried to select film scenes from a large sample (n = 50) in a systematic way, which produced a sample of 37 quality scenes for teaching. Most of the previous studies only described potential areas of interest, without using any statistical analysis^23^^,^^29^ to account for different populations (undergraduate students or residents) with different goals.^29^^,^^30^ Considering this, we looked at one important area of medicine (addictions) and attempted to select material to be used specifically in medical education in this area.

It was also difficult to compare results across different geographical regions. Hence, few definite conclusions can be drawn. Moreover, few studies relating to use of films for teaching about addiction medicine have been conducted. Welsh^31^ used a videotape that was made by combining clips from various commercially available films, along with clips from several television news shows as well as a training film displaying intoxication and withdrawal syndromes. Among second-year medical students, more than 90% of the 89 respondents found the clips helpful in recognizing these syndromes and for appreciating the potential severity of these disorders. Unfortunately, we did not find any film scenes relating to withdrawal. On the other hand, our study provides the opportunity of using many film scenes that present intoxication caused by many drugs, in medical education. Further evaluations on whether the target audiences found the material helpful can be undertaken. In addition, teachers would be able to ascertain whether their students learned more with this technique. However, a note of caution is necessary, in that these films may perpetuate myths about addiction.^10^

Many studies have focused on American cinema,^12^^,^^15^^,^^28^ and they may not be entirely suitable for use within other cultures, since the appropriate cultural context may be missing. The present study has attempted to expand the scientific literature on this field beyond Hollywood. As could be expected, we found that certain Brazilian cultural values were reflected in films, such as Rio de Janeiro’s hillside shantytowns in *Tropa de elite, Cartola, O maior amor do mundo* and *Quase irmãos* (*The elite squad, Top hat, The greatest love of all* and *Almost brothers*); young, wealthy hedonists in *O invasor, Cama de gato* and *Meu nome não é Johnny* (*The invader, Cat’s cradle* and *My name is not Johnny*); and a man who would not say no to liquor in *O homem que desafiou o diabo* (*The man who defied the devil*). Hence, these films can be used appropriately in teaching Brazilian students. Studies on other nationalities’ films could be carried out to look at the country in question or at regional cultural values.

Courses using films in relation to mental illness, within Psychiatry, have been shown elsewhere to be effective and enjoyable for both the students and teachers involved.^30^ Selected scenes can also be used to discuss new substances and new concepts of substance use with students of all professions, especially medicine and psychiatry. One example is the currently increasing use of club drugs, which still have low prevalence of use and dependence.^32^ These substances could be discussed after viewing films such as *Cama de gato* (*Cat’s cradle*) and *O invasor* (*The invader*). For crack, a substance that has increasingly interested Brazilian researchers,^33^^,^^34^ films could be used to discuss some current issues, which include using prostitution as a source of money to purchase crack^34^ and sexually risky behavior among crack users.^33^ Both issues were depicted in *Dois perdidos numa noite suja* (*Two lost in a dirty night*).

Garrison reported^19^ using certain films to portray increased family engagement during psychiatric hospitalizations. Unfortunately, our sample showed hospitalization for patients only in negative terms, such as in scenes from *Bicho de sete cabeças* (*Brainstorm*) and *Meu nome não é Johnny* (*My name is not Johnny*). In *Meu nome não é Johnny*, the film shows the recovery of the main character (*Johnny*) after treatment.

## LIMITATIONS

The selection of films from only one nationality is both a strength and a weakness of the present study. However, this study suggests ways of selecting films in a more robust manner. Even though the internet has facilitated official access to Brazilian films, most of these films have subtitles only in English. It makes access difficult for students and teachers who are not fluent in English or Portuguese. We hope that further studies on other nationalities’ films can fill this gap. It would be worth comparing Brazilian films with an American sample in the future, in order to evaluate whether the use of substances is shown in the same way, in terms of quantity, quality and context, even though cultural values will differ.

## CONCLUSION

Having appropriately and cautiously selected films at hand can provide additional and interesting as well as stimulating starters for discussion of psychopathology and treatment. Numerous psychoactive substances appeared in the films that were evaluated in this study. Because drug use is closely linked to the culture in which an individual is living, only Brazilian cinema can accurately portray the behavior of Brazilians, which is what students must take into account during their training and clinical practice. Moreover, it is possible to reflect on how some of these drug use habits are deeply rooted in some Brazilian groups.
